# Mast cell–neuron axis as a core mechanism in chronic pruritus of atopic dermatitis: from mechanistic insights to therapeutic targets

**DOI:** 10.3389/fimmu.2025.1645095

**Published:** 2025-10-29

**Authors:** Dongdong Li, Yusheng Han, Jingjing Zhou, Huasen Yang, Jing Chen, Hong Liang Tey, Timothy T. Y. Tan

**Affiliations:** ^1^ College of Basic Medical Sciences, Heilongjiang University of Chinese Medicine, Harbin, China; ^2^ School of Chemistry, Chemical Engineering and Biotechnology, Nanyang Technological University, Singapore, Singapore; ^3^ Experimental Teaching & Practical Training Center, Heilongjiang University of Chinese Medicine, Harbin, China; ^4^ Department of Dermatology, Chongqing Traditional Chinese Medicine Hospital, Chongqing, China; ^5^ National Skin Centre, Singapore, Singapore; ^6^ Skin Research Institute of Singapore, Singapore, Singapore; ^7^ Lee Kong Chian School of Medicine, Nanyang Technological University, Singapore, Singapore

**Keywords:** atopic dermatitis, chronic pruritus, mast cell-neuron axis, neuroimmune crosstalk, IL-31 signaling, MRGPRX2, targeted therapy atopic dermatitis, targeted therapy

## Abstract

Chronic pruritus is a defining and therapeutically challenging symptom of atopic dermatitis (AD). Recent advances highlight the mast cell–neuron axis as a central neuroimmune interface orchestrating bidirectional crosstalk between the immune and peripheral nervous systems. Skin mast cells located in close proximity to sensory nerve endings release pruritogenic and neuroregulatory mediators, including histamine, tryptase, and nerve growth factor (NGF), and also modulate IL-31 signaling pathways. These mediators act on neuronal receptors such as IL-31RA, protease-activated receptors 1/2 (PAR-1/2), TrkA, and the adenosine triphosphate (ATP)-gated P2X3 receptor, thereby enhancing neuronal excitability and sensitizing transient receptor potential (TRP) channels (TRPV1, TRPA1). Conversely, sensory neurons release neuropeptides, among which substance P (SP) has been clearly demonstrated to activate Mas-related G protein–coupled receptor X2 (MRGPRX2) on mast cells, inducing non-IgE-mediated degranulation, whereas calcitonin gene-related peptide (CGRP) primarily regulates vascular tone and inflammation, with its direct role in MRGPRX2 activation remaining under investigation. This bidirectional interaction drives a feed-forward itch–inflammation loop. This circuit is further amplified by epidermal barrier dysfunction, microbial dysbiosis, type 2 immune polarization, and neurovascular remodeling. Structural adaptations–including intraepidermal nerve fiber branching and synapse-like mast cell–neuron junctions–provide anatomical substrates for chronic peripheral sensitization. While IL-31RA antagonists such as nemolizumab have demonstrated clinical efficacy, emerging targets like MRGPRX2 and TRPV1/TRPA1 channels offer additional therapeutic avenues but face challenges in translation and safety. Moreover, the P2X3 receptor has been proposed as a potential target for neurogenic itch in AD, but current research remains at an early stage and lacks direct clinical validation, highlighting limitations in its therapeutic development. This review provides a comprehensive mechanistic synthesis of the mast cell–neuron axis in AD-associated pruritus, critically evaluates current and investigational therapies, and explores the potential of multi-target interventions, including traditional Chinese medicine (TCM), for axis-level modulation. These efforts support the advancement of precision therapies targeting neuroimmune circuits in chronic inflammatory dermatoses.

## 1 Introduction

Atopic dermatitis (AD) is a prevalent chronic relapsing inflammatory skin disorder, clinically characterized by intense pruritus, eczematous lesions, and impaired skin barrier function, all of which significantly diminish patients’ quality of life (1, 2). Globally, AD affects more than 200 million individuals, with a rising prevalence observed over the past decade, particularly among children and women (3). Pruritus, the hallmark symptom of AD, serves as a major driver of scratching behavior, local inflammation, and disease chronicity. In moderate to severe cases, particularly in pediatric populations, it often manifests as persistent and treatment-refractory itch that contributes to emotional dysregulation, learning difficulties, and substantial impairments in quality of life (4–6). In the United Kingdom alone, itch-associated sleep disturbance and loss of productivity in AD patients have led to an estimated economic loss exceeding £3.8 billion over a 5-year period (7). Although notable progress has been made in alleviating inflammation through targeted immunomodulatory therapies, the effective control of chronic pruritus remains an unmet clinical challenge (8).

Recent evidence suggests that pruritus in AD is not merely a unidirectional response of sensory neurons to external stimuli, but rather arises from complex neuroimmune interactions that sustain pathological signaling (9). Among these, the mast cell–neuron axis has emerged as a pivotal mechanism that links immune activation with sensory hypersensitivity, providing both anatomical and functional integration between the immune and nervous systems (10). mast cells (MCs), densely distributed in the dermis, are frequently located adjacent to sensory nerve endings, establishing a structural foundation for bidirectional communication within this axis (11). Increasing evidence indicates that through the release of inflammatory mediators and neuropeptides, this axis initiates, amplifies, and maintains itch signaling, forming a self-perpetuating itch–inflammation loop that persists even in the absence of continued external triggers.

This review provides a comprehensive overview of the mast cell–neuron axis in AD-related chronic pruritus, with emphasis on its structural basis, molecular pathways, and translational relevance for targeted therapeutic development.

## 2 Mast cell–neuron axis: a pathological nexus of chronic pruritus

**Figure 1:**
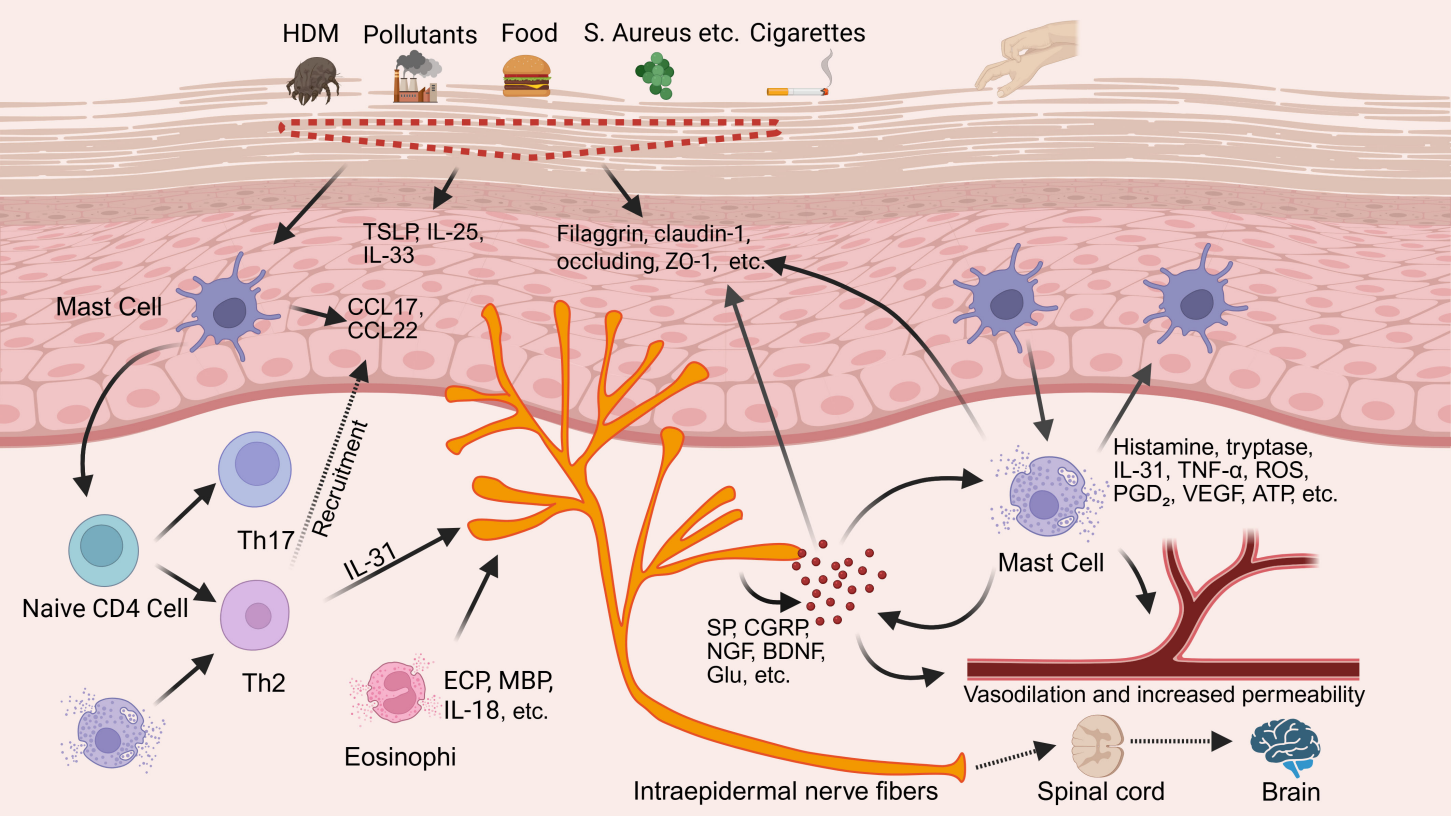
Core role of mast cell–neuron axis in chronic pruritus underlying AD. By BioRender. Note: Tumor necrosis factor alpha, TNF-α; Reactive oxygen species, ROS; Prostaglandin D2, PGD_2_; Vascular endothelial growth factor, VEGF; Adenosine triphosphate, ATP; Eosinophil cationic protein, ECP; Major basic protein, MBP; T helper type, Th; Thymic stromal lymphopoietin, TSLP; Interleukin-, IL-; C-C motif chemokine ligand, CCL; Zonula occludens-1, ZO-1; House dust mite, HDM; Substance P, SP; Calcitonin gene-related peptide, CGRP; Nerve growth factor, NGF; Brain-derived neurotrophic factor, BDNF; Glutamate, Glu.

Chronic pruritus is the hallmark symptom of AD, arising from a multifactorial interplay involving epidermal barrier disruption, microbial dysbiosis, inflammatory mediator accumulation, neuronal sensitization, vascular abnormalities, and neuroplastic remodeling (12). These mechanisms form a dynamic feedback network, in which the mast cell–neuron axis has emerged as a central integrative hub. Bridging neuroimmune signaling pathways, this axis not only initiates itch responses independently but also amplifies and sustains pruritus by integrating inputs from barrier dysfunction, microbial imbalance, and immune activation (13). The overall pathophysiological framework of this axis is illustrated in **Figure 1**.

### 2.1 Crosstalk with epidermal barrier dysfunction

Epidermal barrier dysfunction is a critical initiating factor in the pathogenesis of chronic pruritus in AD, and the mast cell–neuron axis plays an active role not only in responding to external insults but also in perpetuating barrier disruption and impaired repair (14, 15).

On one hand, barrier impairment enhances the skin’s susceptibility to environmental stimuli. Hallmark features include lipid layer depletion and the downregulation of key structural proteins such as filaggrin, claudin-1, loricrin, and Zonula Occludens-1 (ZO-1) (16, 17), thereby facilitating the deeper penetration and immunoneuronal activation by allergens (e.g., house dust mite antigens), bacterial toxins, and other exogenous agents (18). In response to such stimuli, keratinocytes release a series of epithelial alarmins, including thymic stromal lymphopoietin (TSLP), interleukin (IL)-33, and IL-25 (19), which directly activate resident MCs and stimulate sensory neurons, initiating a local neuroimmune cascade (20, 21). On the other hand, once activated, the mast cell–neuron axis exacerbates barrier injury. Mast cell–derived inflammatory mediators such as IL-31 (22, 23) not only enhance neuronal excitability but also act on keratinocytes (24, 25). Through activation of protease-activated receptor 2 (PAR-2), keratinocytes upregulate TSLP, nerve growth factor (NGF), and endothelin-1, thereby facilitating inflammatory cell infiltration, barrier impairment, and aberrant neuro–epidermal interactions that contribute to local inflammation and epidermal abnormalities (26).

Furthermore, neuropeptides released from sensory nerves, such as substance P (SP) and calcitonin gene-related peptide (CGRP), stimulate keratinocytes to produce proinflammatory cytokines including IL-1, IL-6, and TNF (27), and their effects are mediated in part through Ca²^+^ influx and the activation of transient receptor potential (TRP) channels (28), which play important roles in cutaneous inflammation (28, 29).

### 2.2 Crosstalk with cutaneous microbial dysbiosis

As a barrier-associated ecological niche, the skin microbiota plays a pivotal role in maintaining host–microbial homeostasis. In AD, this balance is frequently disrupted, leading to microbial dysbiosis that not only exacerbates local inflammation but also serves as a potential trigger for aberrant activation of the mast cell–neuron axis (12).

Under conditions of barrier disruption or dysbiosis, opportunistic pathogens, most notably *Staphylococcus aureus*, can overcolonize the skin (30). In mouse bone marrow–derived and fetal skin–derived MCs, the δ-toxin secreted by *Staphylococcus aureus* induces non–IgE-dependent degranulation via a Ca²^+^ influx–PI3K signaling pathway, and *in vivo* provokes vascular leakage and eczematous skin lesions; these effects are absent in mast cell–deficient mice but restored upon MCs reconstitution (31). In human LAD2 MCs and HEK293 cells stably transfected with Mas-related G protein–coupled receptor X2 (MRGPRX2), δ-toxin likewise directly engages the MRGPRX2 receptor to trigger degranulation, an effect that can be effectively blocked by the antagonist QWF (32). Collectively, these findings suggest that the δ-toxin–MRGPRX2 axis represents a critical link between cutaneous microbial colonization and MCs activation, and may contribute to Th2-skewed inflammation and pruritus in AD. Thus, microbial dysbiosis acts not only as a passive consequence of barrier dysfunction but also as an active driver of mast cell–neuron axis activation, reinforcing chronic pruritic inflammation in AD.

### 2.3 Crosstalk with immune response networks

In addition to transmitting pruritic signals, the mast cell–neuron axis contributes to immune activation and polarization in AD by modulating the function of various immune cells, thereby participating in the amplification of type 2 inflammation (33).

Upon activation, MCs release a variety of proinflammatory mediators, including IL-4, IL-13, tumor necrosis factor-α (TNF-α), and tryptase. IL-4 and IL-13 promote the differentiation of naïve CD4+ T helper cells (Th0) into T helper 2 cells (Th2) cells and maintain their effector function (34, 35). Tryptase and tumor necrosis factor-alpha (TNF-α) have been shown to activate immune signaling pathways through protease-activated receptor 2 (PAR-2) and tumor necrosis factor receptor 2 (TNFR2), respectively (13). Tryptase activates PAR-2 on endothelial and immune cells, leading to the engagement of downstream ERK/NF-κB signaling cascades and the subsequent release of pro-inflammatory mediators (36). Meanwhile, TNF-α signals through TNFR2 on dendritic cells (DCs), promoting their maturation and enhancing the production of interleukin-12 (IL-12), thereby facilitating Th1 polarization (37). Recent studies also highlight the immunomodulatory role of natural compounds such as diarylheptanoids derived from Curcuma kwangsiensis, which have been found to suppress DC maturation, antigen uptake, and the secretion of IL-6 and IL-12, ultimately inhibiting Th1 and Th17 differentiation. These findings collectively underscore the critical role of DC signaling pathways in shaping adaptive immune responses and maintaining inflammatory homeostasis (38). Moreover, mast cell–derived IL-4 can also activate the STAT6 signaling pathway in dendritic cells (DCs), leading to the upregulation of the chemokines CCL17 and CCL22. These chemokines are crucial for the recruitment of Th2 cells, thereby promoting the establishment of a Th2-skewed immune microenvironment in inflamed skin lesions (39). IL-31 secreted by activated Th2 cells binds to IL-31RA on sensory neurons, activating STAT3 signaling and inducing the release of neuropeptides such as CGRP, which contributes to neurogenic inflammation and modulation of local immune responses (24, 40). In addition, upon recruitment to inflamed skin, eosinophils release cytotoxic granules containing major basic protein (MBP) and eosinophil cationic protein (ECP) (41), which can directly act on peripheral sensory nerve terminals, alter membrane potential, and induce neuronal hyperexcitability, contributing to pruritus and neurogenic inflammation (42). Although IL-4 and IL-13 do not directly activate sensory neurons, they upregulate the expression of transient receptor potential (TRP) channels and related gene programs, thereby enhancing neuronal sensitivity to pruritogens and promoting peripheral sensitization (43, 44).

### 2.4 Crosstalk with the neurovascular system

A bidirectional regulatory relationship exists between the mast cell–neuron axis and the local cutaneous vasculature, forming a key conduit for inflammatory spread and peripheral neural sensitization (45). Under chronic inflammatory conditions, the neurovascular unit undergoes structural remodeling, characterized by increased sensory nerve fiber density and impaired endothelial barrier function, which strengthens neuro–immune–vascular coupling and provides a histopathological foundation for the persistence and therapeutic resistance of chronic pruritus in AD (15, 26).

Upon activation, MCs rapidly release a repertoire of vasoactive mediators, including histamine, ATP, prostaglandin D_2_ (PGD_2_), leukotrienes (e.g., LTC_4_), vascular endothelial growth factor (VEGF), and TNF-α (46, 47). These factors act on endothelial cells to induce vasodilation, increase vascular permeability, and trigger neurogenic vasoreflexes (48, 49). Experimental evidence demonstrates that mast cell–derived mediators can directly act on receptors of sensory neurons, enhancing the sensitivity of peripheral nerve endings to inflammatory signals and thereby sustaining the persistent transmission of itch (50, 51).Conversely, sensory neurons, upon activation, release neuropeptides such as SP and CGRP, which act directly on vascular smooth muscle cells and endothelial cells to induce neurogenic vasodilation and plasma extravasation (52, 53). This process reinforces the establishment of a neurovascular–immune coupling network. Notably, activation of transient receptor potential vanilloid 1 (TRPV1) and transient receptor potential ankyrin 1 (TRPA1) channels enhances CGRP release from sensory nerve endings (54), which has been shown to promote capillary dilation, sustain neurogenic inflammation, and activate MCs, thereby amplifying local itch and inflammation (52, 55).

Importantly, the synergistic interaction between vascular-derived factors such as VEGF and neuropeptides has been widely observed in various dermatological disease models. These findings suggest that cutaneous vasculature is not merely a passive route for immune cell trafficking but also serves as an active amplifier of pruritic signaling (56, 57).

## 3 Bidirectional crosstalk between MCs and intraepidermal nerve fibers

**Figure 2:**
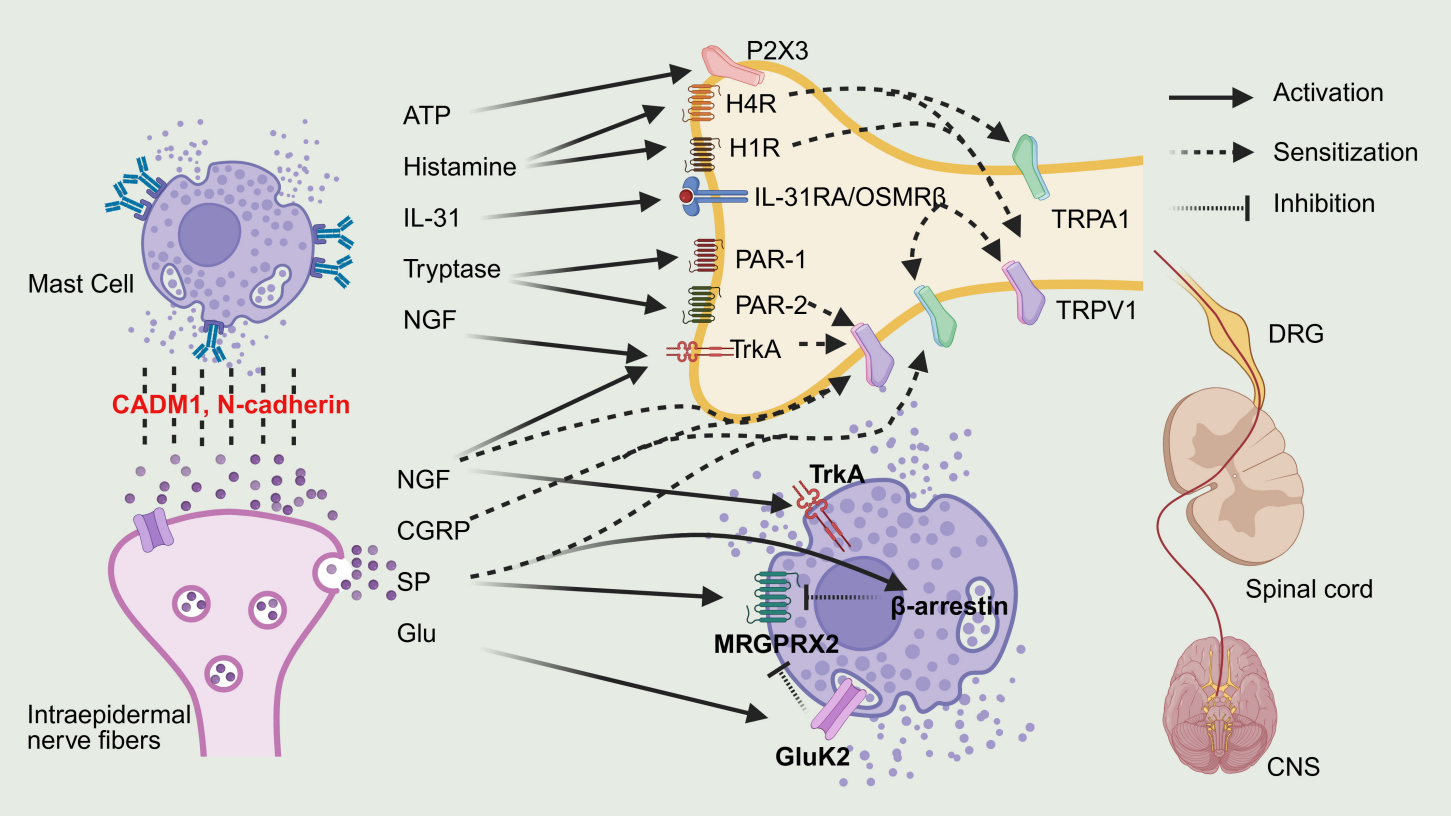
Bidirectional crosstalk between MCs and sensory neurons. By BioRender. Note: Interleukin-31, IL-31; Interleukin-31 Receptor A, IL-31RA; Oncostatin M receptor beta, OSMRβ; Nerve growth factor, NGF;Tropomyosin receptor kinase A, TrkA; Substance P, SP; Calcitonin gene-related peptide, CGRP; Glutamate, Glu; Glutamate Ionotropic receptor kainate type subunit 2, GluK2; Mas-related G protein-coupled receptor X2, MRGPRX2; Protease-activated receptor-2/-1, PAR-2/1; Transient receptor potential ankyrin 1, TRPA1; Transient receptor potential vanilloid 1, TRPV1; Histamine receptor H1/H4, H1R/H4R; Central nervous system, CNS; Dorsal root ganglion, DRG.

The bidirectional communication between mast cells and sensory neurons and their signaling pathways are illustrated in **Figure 2**.

### 3.1 Structural basis of MCs and intraepidermal nerve fibers

#### 3.1.1 MCs

MCs are tissue-resident immune cells derived from hematopoietic stem cells in the bone marrow. They are broadly distributed across barrier tissues such as the skin, respiratory tract, and gastrointestinal tract, with particularly high densities in the dermis (58). Traditionally, skin MCs are classified as the connective tissue type (MCTC), characterized by the presence of both tryptase and chymase in their secretory granules, in contrast to the mucosal type (MCT), which contains tryptase but lacks chymase (59). Recent advances in single-cell transcriptomic profiling, however, have revealed that skin MCs are not a homogeneous MCTC population. Instead, they comprise transcriptionally diverse subsets. In mice, skin MCs are almost exclusively MrgprB2^+^ connective tissue–type cells of embryonic origin that persist independently of bone marrow renewal (60), whereas in humans, at least four distinct clusters (MC1–MC4) are enriched in the skin, including MC1 as a dominant subset with distinct effector programs and spatial associations (61). These findings highlight that skin MC heterogeneity extends far beyond the classical protease-based dichotomy, reflecting developmental origin, tissue niche, and disease-specific adaptations. In the skin, MCs are commonly localized near blood vessels, lymphatic vessels, and peripheral sensory nerve endings, forming a highly dynamic neuroimmune interface that anatomically supports local interactions between inflammatory and neural signals (62, 63). In active lesions of AD, the number of skin MCs is significantly elevated (10, 64). Mechanistically, this focal accumulation is thought to result from the synergistic effects of cytokines such as IL-4, IL-33, and TSLP, along with chemokines such as CCL17 and CCL22 within the inflamed tissue microenvironment (65, 66).

In terms of receptor expression, MCs are traditionally characterized by high levels of FcϵRI, the high-affinity Immunoglobulin E (IgE) receptor that mediates type I hypersensitivity responses and plays a key role in initiating allergic inflammation in AD (67, 68). In addition, skin MCs selectively express MRGPRX2 (69), which recognizes neurogenic ligands such as SP, CGRP, and neurokinin A (33, 70). This dual receptor repertoire enables MCs to respond not only to IgE-mediated stimuli but also to neuropeptide signaling, thereby contributing to both allergic and non-IgE-dependent neurogenic inflammation (71). Together, these properties establish MCs as a central component of the neuroimmune interaction network in AD.

#### 3.1.2 Intraepidermal nerve fibers

On the neuronal side, the skin is primarily innervated by unmyelinated C-fibers and thinly myelinated Aδ-fibers, which play essential roles in itch perception and transmission (72, 73).

Early investigations using 2-dimensional methods to study the histological and ultrastructural features of AD have yielded conflicting results. Some studies have reported marked neural remodeling in lesional skin, including axonal hypertrophy, increased axon numbers, and a significant elevation in intraepidermal nerve fiber density (IENFD) (74, 75). However, other studies have arrived at opposite conclusions (76, 77). With the implementation of advanced 3-dimensional imaging modalities, subsequent research has increasingly demonstrated that chronic mechanical injury, barrier dysfunction, and local inflammation contribute to a paradoxical reduction in IENFD within AD lesions (78, 79). Although an increased IENFD is commonly observed in acute or subacute lesions of AD, this phenomenon is thought to be closely associated with elevated levels of NGF and reduced expression of the axon-repelling molecule Semaphorin 3A (Sema3A) (80, 81). However, as the disease progresses into a chronic phase, the expression of Sema3A gradually increases, potentially inhibiting further neurite outgrowth and contributing to a reduction in cutaneous nerve fiber density (82).Recent evidence highlights that, rather than total nerve fiber density alone, qualitative alterations in specific subsets of sensory fibers such as increased branching, convoluted trajectories, and terminal swellings represent key morphological features of peripheral sensitization (82, 83). These structural alterations decrease the activation threshold of sensory neurons and bring them into closer proximity to environmental stimuli, thereby increasing susceptibility to light touch, allergens, and chemical irritants and facilitating itch responses (5). Notably, neural remodeling in AD appears to be at least partially reversible. This pathological feature is further substantiated by the reversal of nerve fiber alterations in lesional skin observed after dupilumab therapy (84).

Intraepidermal nerve fibers in AD can be subdivided into functionally distinct subtypes based on their molecular markers, which define their roles in histamine-independent pruritic signaling and the chronicity of itch. According to the expression patterns of the Mas-related G protein–coupled receptor (Mrgpr) family, intraepidermal nerve fibers in AD can be categorized into MrgprA3^+^ (85), MrgprX1^+^ (86), and MrgprX2^+^ (33, 87)subtypes. These fibers primarily mediate non-histaminergic itch by responding to exogenous stimuli or activating immune cells such as MCs, thereby inducing both acute and chronic pruritic responses and contributing to neuroimmune interactions within the AD microenvironment (88).

From the perspective of transient receptor potential (TRP) channel expression, TRPV1^+^ and TRPA1^+^ fibers represent key pathways for itch transmission in AD (89, 90). These ion channels detect thermal, mechanical, and chemical stimuli and are sensitized by inflammatory mediators such as IL-31, thereby increasing neuronal excitability and amplifying pruritic signaling (91).

In addition to TRP channels, ATP-gated P2X3 receptors represent another important class of ion channels expressed by specific subsets of intraepidermal sensory fibers. Notably, MrgprA3^+^ pruriceptive neurons, which are enriched in lesional skin of AD, exhibit high levels of P2X3 expression as revealed by transcriptional profiling (92). These receptors mediate rapid depolarization in response to extracellular ATP and are further sensitized by neurotrophic factors such as NGF and CGRP (93, 94).

### 3.2 Mast cell–mediated neuronal excitation and sensitization

In lesional skin of patients with AD, MCs release a repertoire of bioactive mediators, including histamine, IL-31, tryptase, and NGF, at markedly elevated levels, reflecting an active state of the MC–neuron axis in the pathogenesis of pruritus (95, 96).

Histamine induces acute itch by activating H1 and H4 receptors on sensory neurons (97, 98). IL-31 signals through a heterodimeric receptor complex composed of IL-31RA and oncostatin M receptor β (OSMRβ), eliciting chronic itch and enhancing TRPV1 activity (91). Notably, IL-31RA expression is regulated by STAT3 and may persist even after the resolution of inflammation, suggesting the existence of a ‘neuronal itch memory’ (40). Tryptase primarily activates PAR-2, and to a lesser extent PAR-1, on sensory neurons, inducing intracellular calcium influx and triggering downstream signaling cascades that amplify neuronal excitability and promote local inflammation (99, 100). NGF binds to its high-affinity receptor TrkA, promoting axonal growth cone formation and epidermal neurite extension, thereby increasing intraepidermal nerve fiber density, enhancing peripheral sensitivity, and aggravating itch perception (101, 102).

In addition to direct neuronal activation, MC-derived mediators further contribute to neuronal sensitization by increasing the activity of membrane-bound TRP channels, particularly TRPA1 and TRPV1, which lower the activation threshold of neurons and establish a state of peripheral hyperexcitability (40, 91, 103). Among these, TRPA1 is particularly active in inflamed skin and has been implicated in both pruriceptive transduction and immune amplification (104, 105). TRPV1, on the other hand, has been demonstrated to mediate MC-dependent itch in AD models, and its pharmacological blockade significantly reduces scratching behavior and alleviates local inflammation (90).

Beyond the well-established roles of histamine, IL-31, and neuropeptides in sensory modulation, purinergic signaling mediated by ATP has recently emerged as a critical complementary pathway in the sensitization of the mast cell–neuron axis in AD, with particular attention to the P2X3 receptor. Single-cell transcriptomic analyses of dorsal root ganglia have identified pruriceptive neuron subsets co-expressing P2rx3 and itch-related markers, supporting P2X3 as a molecular signature in sensory neurons involved in inflammatory skin diseases (106). Experimental studies have demonstrated that P2X3 regulates scratching behavior in murine models of itch, and pharmacological inhibition of P2X3 significantly attenuates symptoms, highlighting its pivotal role in pruritic signal transduction, despite the absence of direct evidence in AD-specific models (92). Furthermore, P2X3 activation has been shown to potentiate IgE-dependent MCs degranulation, thereby amplifying immune-mediated inflammation and supporting its involvement in allergic disease contexts (107).

P2X3 receptors are also highly responsive to neurotrophic factors such as NGF and CGRP, both of which are elevated in inflamed skin and capable of enhancing P2X3 expression while lowering the threshold for neuronal activation, thereby promoting a state of hyperexcitability (108). Given that P2X3 is also expressed in MCs, this receptor may contribute to a bidirectional amplification loop between immune and sensory cells, driving sustained neuroinflammation and chronic itch (109). Although direct experimental validation in AD remains limited, these findings collectively provide important mechanistic insight into the potential involvement of P2X3 in the regulation of the mast cell–neuron axis.

### 3.3 Neuronal feedback loops driving MCs reactivation

Sensory neurons not only mediate itch perception but also play a pivotal role in amplifying skin inflammation through active neuroimmune interactions. Neuropeptide-expressing fibers, including those positive for SP and CGRP, interact with immune cells such as MCs to initiate neurogenic inflammation and promote the release of type 2 cytokines like IL-4 and IL-13, thereby exacerbating both cutaneous inflammation and pruritus (24, 29). In parallel, fibers expressing the cytokine receptor IL-31RA respond to IL-31 secreted by T cells by driving neurite outgrowth and branching, a structural remodeling process that increases cutaneous sensitivity to mild stimuli and represents a key mechanism underlying chronic itch in AD (40, 110).

A central feature of chronic itch in AD is sustained MCs activation mediated through MRGPRX2, which is significantly upregulated in MCs within active lesions (35). SP released from activated intraepidermal nerve fibers binds to MRGPRX2 on MCs, triggering non-IgE-mediated degranulation and initiating a positive neuroimmune feedback loop (24, 111). At the signaling level, SP–MRGPRX2 engagement activates calcium-dependent degranulation pathways and drives nuclear translocation of lysyl-tRNA synthetase (LysRS), leading to activation of the MITF signaling axis and enhanced inflammatory mediator production (112). Neurotrophic factors further strengthen this loop. NGF and BDNF promote MCs migration toward the superficial dermis and enhance their responsiveness through TrkA-mediated signaling (29, 113). Concurrently, NGF binds to TrkA on sensory neurons, promoting axonal outgrowth, intraepidermal extension, and increased branching complexity, ultimately enhancing cutaneous neuroresponsiveness and contributing to chronic itch (82, 101, 114).

Importantly, neuronal regulation of MCs is not exclusively proinflammatory. Recent findings suggest that certain non-peptidergic sensory neurons release glutamate, which acts on GluK2 receptors expressed by MCs to downregulate mice MrgprB2 expression and suppress degranulation (115). In addition, SP exhibits biased agonism by recruiting β-arrestin, which modulates MRGPRX2 internalization and signal regulation (116). These mechanisms highlight the existence of a potential negative feedback circuit that fine-tunes neuroimmune activation in AD.

## 4 Structural and functional remodeling in chronic pruritus

### 4.1 Plasticity of mast cell–neuron axis

Chronic pruritus in AD is not merely the result of prolonged immune activation but also arises from sustained remodeling of the peripheral neuroimmune architecture (117). The mast cell–neuron axis, located within the dermis as a critical amplification unit, plays a central role in initiating and maintaining itch signaling (50). Studies have shown that MCs tend to accumulate around peripheral sensory nerve terminals (118), and their density is markedly increased in lesional AD skin (58), where they frequently remain in a highly degranulated state (35, 119).

A potential anatomical basis may underlie the amplification of chronic itch. In addition to molecular amplification, structural adaptations further reinforce this pathway. Studies have shown that under inflammatory conditions, MCs can undergo remodeling, forming tighter synapse-like structures with sensory neurons and thereby contributing to peripheral neural sensitization (45, 119). In AD lesions, these synapse-like junctions are mediated by adhesion molecules such as cell adhesion molecule 1 (CADM1) and N-cadherin, facilitating highly efficient local signal transmission (118). Cryo-electron microscopy and confocal imaging have further confirmed the presence of such structures, demonstrating that they enable efficient, short-range signal exchange between cells (29, 119). These structural interactions were initially described in models of asthma and neuroinflammation (120), and similar patterns have recently been observed in the lesional skin of murine AD models, where MCs tend to cluster near nerve terminals (121, 122). Recent experimental data further indicate that pharmacological or genetic disruption of these synapse-like MC–neuron contacts alleviates itch behavior and reduces peripheral sensitization in murine AD models, underscoring their functional relevance in chronic neuroinflammation (93).

From the neuronal perspective, intraepidermal nerve fibers in AD can be subdivided into functionally distinct subsets that mediate histamine-independent itch by responding to external stimuli or by engaging immune cells such as MCs, thereby contributing to both acute and chronic pruritus and shaping neuro–immune interactions in the lesional microenvironment (123). In addition, fibers expressing IL-31RA respond to IL-31 secreted by T cells by promoting neurite outgrowth and branching, a structural remodeling process that enhances cutaneous sensitivity to mild stimuli and represents a key mechanism underlying chronic itch (33, 90) (124).

### 4.2 Peripheral sensitization

A defining feature of peripheral sensitization is the lowering of the neuronal activation threshold (125), which refers to the minimal membrane depolarization required to initiate an action potential in sensory neurons. Under homeostatic conditions, this threshold prevents excessive neuronal firing in response to innocuous stimuli (126). However, in the context of chronic inflammation, pruritogenic mediators and neurotrophic factors can significantly reduce this threshold, rendering sensory fibers hyperexcitable and responsive to otherwise non-pruritic inputs (5, 127).

Among the molecular determinants of this lowered threshold, TRP channels have been extensively studied. Transient receptor potential (TRP) channels further contribute to this sensitization process. TRPV1^+^ and TRPA1^+^ fibers detect thermal, mechanical, and chemical stimuli and are sensitized by inflammatory mediators such as IL-31 (91), resulting in heightened neuronal excitability and amplification of itch signaling (128). Under inflammatory conditions, TRPV1 expression is significantly upregulated (91), and its activation enhances neuronal responsiveness to pruritogens while promoting the release of neuropeptides such as SP and CGRP (124, 129, 130). These neuropeptides interact with MCs to initiate neurogenic inflammation and stimulate the release of type 2 cytokines such as IL-4 and IL-13, thereby exacerbating cutaneous inflammation and pruritus (35) (131).

In addition to TRP channels, purinergic signaling via P2X3 receptors also plays a critical role in neuronal sensitization under inflammatory conditions (132). P2X3 is an ATP-gated cation channel highly expressed in itch-selective neurons and has been shown to mediate scratching behavior in murine models of pruritus (133). In AD models, upregulation of P2X3 receptors has been demonstrated to mediate itch-associated scratching (133). Moreover, NGF, which is elevated in AD skin, has been reported to enhance neuronal sensitization partly by potentiating P2X3 receptor function via rapid PKC activation (134, 135), a mechanism highlighted in recent reviews on peripheral itch sensitization in AD (5). NGF has been shown to upregulate P2X3 receptor transcription and enhance its trafficking to the neuronal membrane, thereby increasing surface availability and amplifying ATP-evoked responses (136). These molecular changes heighten neuronal responsiveness to extracellular ATP. Moreover, NGF stimulation induces spontaneous membrane potential fluctuations and intracellular calcium influx—hallmarks of increased excitability—which further amplify P2X3-mediated signaling (137). Together, these effects position P2X3 as a central mediator of NGF-driven peripheral sensitization and hyperexcitability in sensory neurons.

### 4.3 neuroimmune feedback driving chronic itch persistence

Chronic itch in AD is not merely a symptom but a dynamic driver of disease progression. Sensory neurons, particularly C-type fibers, play an active role in sustaining inflammation. Upon exposure to mechanical or allergen-derived stimuli as well as proinflammatory cytokines, these neurons release neuropeptides, such as SP and CGRP (69), which accumulate in lesional skin and amplify local immune responses (119).

Scratching, a behavioral consequence of itch, further perpetuates this cycle. Mechanical stimulation enhances TRPV1 activation in peripheral sensory neurons, leading to the release of neuropeptides such as SP and CGRP, thereby perpetuating the itch-scratch cycle through mechanical stimulation (90). In human MCs, SP, particularly in the presence of interleukin-33 (IL-33), enhances IL-31 gene expression and secretion via MRGPRX2 activation, independent of classical degranulation pathways (22). In parallel, TRPV1^+^ nociceptive neurons release neuropeptides such as SP which activate MrgprB2 the murine ortholog of human MRGPRX2 on adjacent MCs leading to non-IgE-mediated degranulation and the release of inflammatory mediators including tryptase thereby promoting type 2 inflammation (35, 119). These mediators, in turn, act via IL-31RA and TRPV1 to sustain neuronal hyperexcitability, creating a self-reinforcing loop of scratching–release–reactivation (40, 91).

At the molecular level, this cycle forms a closed mediator–receptor–remediator feedback circuit. Neuropeptide-induced MCs degranulation leads to release of pruritogenic mediators, which further sensitize neurons and promote additional neuropeptide release (22, 24). Non-peptidergic sensory neurons, particularly MrgprD^+^ subsets, have been shown to tonically release glutamate, thereby restraining MCs hyperresponsiveness through regulation of Mrgprb2 expression (115). Building on this finding, recent evidence demonstrated that glutamate acts via GluK2 receptors on MCs to suppress Mrgprb2/MRGPRX2 expression and inhibit degranulation (138). Together, these studies delineate a previously unrecognized neuroimmune feedback circuit in which neuronal glutamate provides a negative feedback brake to restrain MCs hyperactivation in cutaneous inflammation.

## 5 Therapeutic targets in the mast cell–neuron axis

An overview of the major molecular targets and related therapeutic agents is summarized in **Table 1**.

**Table 1:** Therapeutic targets in the mast cell–neuron axis.

Drug name	Targets	Development stage	Organisms	Outcome	References
Nemolizumab (CIM331)	IL-31RA	Approved	Homo sapiens (AD patients)	↓pruritus (rapid & sustained), ↓EASI, ↑EASI-75, ↑IGA success, ↓sleep disturbance, ↓DLQI, ↓proinflammatory biomarkers (CCL20, CCL22, CCL27, VEGF); well tolerated with mainly injection-site reactions and nasopharyngitis	(141–145)
Lokivetmab(ZTS-00103289)	IL-31	Approved	Canis lupus familiaris (AD patients)	VAS↓, CADESI-03↓, rapid onset (Day 1), sustained up to 1 month; noninferior to ciclosporin for pruritus but less effective for skin lesions, long-term control (>70% at 9 months), well tolerated with low immunogenicity	(147–150)
EVO756	MRGPRX2	*In vitro*	Primary human skin-derived MCs, LAD2 cells, ROSA cells, CHO-MRGPRX2 cells	↓histamine, ↓CD63, ↓IL-8, ↓TNF-α, ↓MRGPRX2 internalization, ↓transcriptional activation, ↔IgE–FcϵRI response	(157, 158)
EP262	MRGPRX2	*In vitro*/*In vivo*/ongoing clinical development	LAD2 cells, primary human MCs,human MRGPRX2 knock-in mice	↓β-hex, tryptase, histamine, IL-6, TNF-α, ↓MCs degranulation, ↓vascular permeability, ↔IgE–FcϵRI response	(159, 160)
GE1111	MRGPRX2	*In vitro*/*In vivo*	LAD2 cells, primary human skin-derived MCs, HaCaT keratinocytes, RAW264.7 macrophages, mice	↓TSLP, IL-13, IL-1β, TNF-α, MCP-1, ↑claudin-1; ↓periostin, ↓skin thickening, erythema, scaling, restored skin integrity	(161)
C9	MRGPRX2	*In vitro*/*In vivo*	LAD2 cells, RBL-2H3 MRGPRX2-transfected cells, primary human skin MCs, mouse peritoneal MCs	↓MRGPRX2 basal activity, ↓SP- and C48/80-induced degranulation, ↓Ca²^+^ signaling, ↓β-arrestin recruitment, ↔IgE–FcϵRI response, ↔MrgprB2 response	(162)
HC-030031	TRPA1	*In vitro*/*In vivo*	RAW264.7 macrophages, mice	↓skin inflammation, ↓dermatitis score, ↓ear thickness, ↓pruritus, ↓epidermal hyperplasia, ↓MCs & macrophage infiltration, ↓IL-4, IL-13, TNF-α, TRPA1 antagonist HC-030031 replicated effects	(105)
Carboxamide TRPA1 antagonists	TRPA1	*In vitro*/*In vivo*	CHO cells, CHO-hTRPA1 cells, cavia porcellus	↓TRPA1-mediated Ca²^+^ influx, ↑TRPA1 selectivity, ↓skin flare in topical cinnamaldehyde model	(163)
Asivatrep(PAC-14028)	TRPV1	Clinical trials (Phase II and III)	Homo sapiens (AD patients)	IGA↓, EASI↓, VAS↓, improved pruritus-related sleep disturbances	(165, 166)
Zhenxin Anshen formula	TRPV1/TRPA1	*In vitro*/*In vivo*	HaCaT keratinocytes, mice	↓SCORAD; ↓spontaneous scratching; ↑skin structure integrity; ↓serum IgE, ↓IL-4, ↓IL-5, ↓IL-13, ↓TSLP; ↓GRPR, ↓TRPV1, ↓TRPA1; ↓IL-6, ↓TNF-α; ↓Ca²^+^ influx in DRG neurons	(167)
Siglec-8 monoclonal antibody	Siglec-8	*In vitro*/*Ex vivo*/*In vivo*	human AD skin biopsies, human MCs, Siglec-8 transgenic mice	↓MRGPRX2 ligand-induced inflammation & pruritus, ↓MCs activation & degranulation, ↓CPA3, ↓IL-8, ↓IL-13, ↓IL-31, ↓CCL17	(95)
SYM2081	GluK2	*In vitro*/*Ex vivo*/*In vivo*	Mouse peritoneal MCs, human skin explants, mice	↓MrgprB2 expression, ↓MCs degranulation, ↓MCs proliferation (↓Ki-67, ↓BrdU), ↓chymase, ↓β-hex, ↓IL-1β, ↓CCL2, ↓IL-27, ↓skin lesion thickness & infiltration, ↓skin inflammation	(138)
Synta66	Orai1/2/3 (CRAC channels)	*In vitro*/*In vivo*	LAD2 cells, human MCs, mouse peritoneal MCs, mice	↓Ca²^+^ influx, ↓MC degranulation (β-hex, LAMP-1), ↓TNF-α, ↓IL-8, ↓CCL3; ↓ERK1/2 & Akt activation, ↓ear vascular leakage, ↓neutrophil recruitment	(173)
DS-2741a	ORAI1	*In vitro*/*In vivo*	Human T cells, human MCs, human ORAI1 knock-in mice	↓ MCs degranulation (β-hex), ↓ T cell activation, ↓ skin inflammation in HDM-induced dermatitis (human ORAI1 knock-in mice)	(174)
Myricetin	CD300f	*In vitro*/*In vivo*	LAD2 cells, mice	↓MCs degranulation, ↓vascular permeability, ↓β-hex & histamine release, ↓TNF-α, ↓IL-8, ↓MCP-1, ↓Ca²^+^ influx, ↓phosphorylation of PLCγ1, AKT, p38, ERK1/2	(176)
Dehydroandrographolide(DA)	CD300f	*In vitro*/*In vivo*	LAD2 cells, mice	↓MC degranulation, ↓β-hex & histamine release, ↓Ca²^+^ influx, ↑SHP1/2 phosphorylation (CD300f-dependent), ↓vascular dilation, erythema, and serum cytokines (unspecified), attenuated pseudo-allergic reactions	(175)
Celastrol	MRGPRX2/ORAI1/ORAI2 axis	*In vivo*	Mice	↓scratching behavior, ↓skin thickening, ↓MCs numbers & inflammatory infiltration, ↓serum histamine, ↓MRGPRX2, ORAI1/2 expression, ↓TNF-α, ↓IL-6, ↓IL-1β, and ↓IL-31	(177)

Notice: Downregulation, ↓; Upregulation, ↑; No effect, ↔; Interleukin-31 receptor A, IL-31RA; Transient receptor potential ankyrin 1, TRPA1; Transient receptor potential vanilloid 1, TRPV1; Glutamate ionotropic receptor kainate type subunit 2, GluK2; Calcium release–activated calcium channel protein, ORAI; Mast cells, MCs; Human mast cell line derived from peripheral blood progenitors, LAD2 cells; Rat basophilic leukemia–derived mast cell line, ROSA cells; Chinese hamster ovary cells stably expressing human MRGPRX2, CHO-MRGPRX2 cells; Spontaneously immortalized human keratinocyte line, HaCaT keratinocytes; Murine macrophage line derived from Abelson leukemia virus–induced tumor, RAW264.7 macrophages; Rat basophilic leukemia cell line with mast cell–like properties, RBL-2H3 mast cells; Chinese hamster ovary cells, CHO cells; Chinese hamster ovary cells stably expressing human TRPA1, CHO-hTRPA1 cells; Eczema Area and Severity Index, EASI; Investigator’s Global Assessment, IGA; Dermatology Life Quality Index, DLQI; Visual Analog Scale, VAS; Canine Atopic Dermatitis Extent and Severity Index-03, CADESI-03; SCORing Atopic Dermatitis, SCORAD; C-C motif chemokine ligand, CCL; Vascular endothelial growth factor, VEGF; Interleukin-, IL-; Tumor necrosis factor-, TNF-; Thymic stromal lymphopoietin, TSLP; Monocyte chemoattractant protein-1, MCP-1; Mas-related G protein–coupled receptor X2, MRGPRX2; Transient receptor potential ankyrin 1, TRPA1; Gastrin-releasing peptide receptor, GRPR; Transient receptor potential vanilloid 1, TRPV1; Mas-related G protein–coupled receptor B2, MrgprB2; Substance P, SP; Cluster of differentiation 63, CD63; Carboxypeptidase A3, CPA3; β-hexosaminidase, β-hex; Bromodeoxyuridine, BrdU; Antimicrobial peptide-1, AMP-1; Extracellular signal–regulated kinase 1/2, ERK1/2; House dust mite, HDM; Phospholipase C-γ1, PLCγ1; Protein kinase B, AKT; p38 mitogen-activated protein kinase, p38; Src homology region 2 domain–containing phosphatase-1/2, SHP1/2; Dorsal root ganglion, DRG.

### 5.1 IL-31/IL-31RA: a clinically validated target

IL-31, a prototypical neuroimmune cytokine secreted by Th2 cells, MCs, and eosinophils, is one of the most extensively validated itch mediators within the mast cell–neuron axis (24). It exerts its pruritogenic effects through a heterodimeric receptor complex composed of IL-31RA and OSMRβ, which is expressed on cutaneous sensory neurons. Receptor activation has been shown to upregulate TRPV1 and TRPA1 channels, thereby inducing a state of neuronal hyperexcitability and establishing a chronic pruritus circuit characterized by a low activation threshold and high firing frequency (91).

In lesional skin of patients with AD, IL-31RA expression is significantly elevated and correlates positively with subjective pruritus scores, such as the Peak Pruritus Numerical Rating Scale (PP-NRS) (91). Notably, IL-31RA expression is regulated by STAT3 within sensory neurons and remains elevated even after clinical resolution of inflammation, suggesting a pruritic memory mechanism that enables sustained neuronal activation independent of primary inflammatory cues (40). Mechanistic studies further indicate that IL-31 not only acts on neurons but also modulates the immune microenvironment, for example, by inducing CGRP release to negatively regulate Th2 responses. These findings imply that blockade of IL-31RA may carry the risk of immune rebound (24, 139).

Nemolizumab, a humanized immunoglobulin G2(IgG2) monoclonal antibody targeting IL-31RA, was specifically developed to inhibit IL-31 signaling within the mast cell–neuron axis and entered clinical development in 2016 (140). Subsequent clinical studies have consistently demonstrated its efficacy in alleviating moderate-to-severe AD–associated pruritus.

In a phase III randomized controlled trials (RCT) in adults with moderate-to-severe AD, nemolizumab with topical therapy significantly improved pruritus and skin lesions. At 16 weeks, the nemolizumab group had a 42.8% reduction in pruritus VAS score vs. 21.4% in the placebo group. The eczema area and severity index (EASI) score decreased by 45.9% in the nemolizumab group versus 33.2% in the placebo group. Nemolizumab also led to significant improvements in sleep disturbances (55% of patients achieved ISI ≤7 vs. 21% in the placebo group) and daily functioning (40% achieved DLQI ≤4 vs. 22% in the placebo group), indicating enhanced quality of life. Additionally, some patients in the nemolizumab group reported significant pruritus relief as early as day 2 of treatment (141). Long-term follow-up studies have shown that these benefits are durable, with efficacy maintained up to 68 weeks, EASI improvement exceeding 70%, and continued decline in pruritus scores, supporting its potential for chronic disease control (142). In the recent Phase III RCTs ARCADIA 1 and ARCADIA 2, nemolizumab plus standard topical therapy achieved a ≥4-point reduction in PP-NRS scores at week 16 in 43–46% of patients versus 18–19% with placebo. Moreover, 28–31% of nemolizumab-treated patients reached a PP-NRS score <2 (indicative of near-complete itch relief), compared with 11% in the placebo arm. Notably, by week 1, 5–7% of patients receiving nemolizumab had already achieved a ≥4-point decrease in PP-NRS, versus <1% with placebo. Improvements were also noted in sleep quality, EASI-75 response, and Investigator’s Global Assessment (IGA) scores, supporting its broad therapeutic benefit (143). These improvements were accompanied by downregulation of inflammatory biomarkers (CCL20, CCL22, CCL27, VEGF), suggesting systemic modulation of the neuroimmune axis (144). The Nemolizumab-JP04 trial extended these findings to pediatric patients aged 6–12 years, showing significant reductions in pruritus NRS and improvements in EASI at week 16, along with better quality of life outcomes including sleep and caregiver burden (145).

With respect to safety, nemolizumab has demonstrated a favorable profile across age groups. Common short-term adverse events included injection site reactions, mild conjunctivitis, and upper respiratory tract infections, all occurring at rates below 5%. No serious drug-related adverse events were reported. Long-term follow-up data confirm that nemolizumab is not associated with cumulative toxicity, immunosuppression, or neurological adverse effects over a 68-week treatment period (142, 146). In adolescents and children, the adverse event profile was consistent with that observed in adults, predominantly mild to moderate in severity and not affecting treatment adherence (144, 145).

Lokivetmab, a monoclonal antibody against IL-31 approved for the treatment of canine AD, provides interspecies validation of the IL-31–neural axis in pruritus. Clinical studies have shown that lokivetmab can significantly relieve itch within 24 hours of administration, with a 2 mg/kg dose maintaining efficacy for approximately 1 month and reducing pruritus visual analog scale (PVAS) scores by more than 50% (147, 148). Its antipruritic efficacy was comparable to that of cyclosporine, although it was less effective for skin lesion resolution, and it was associated with fewer adverse effects (149). Long-term safety studies have not identified any significant immunosuppressive or organ-specific toxicity (150).

A number of patents have been filed for anti-IL-31 antibodies, including IgG4 variants specifically designed to block IL-31 binding to its receptor complex (151). However, aside from nemolizumab, no human IL-31–targeting antibodies have yet completed phase II clinical trials.

### 5.2 MRGPRX2: an emerging pruritogenic target

MRGPRX2 is a member of the G protein-coupled receptor (GPCR) superfamily and functions as a critical signaling hub that links extracellular stimuli to intracellular responses (152, 153). Evidence from both clinical samples and animal models indicates that MRGPRX2 in humans and its murine ortholog MrgprB2 are positively correlated with inflammation severity (35), contribute to sensory neuron activation and scratching behavior (154), and mediate non-IgE-dependent inflammatory responses in AD models (155), highlighting their potential as functional therapeutic targets. In recent years, drug discovery efforts have focused on small-molecule antagonists that inhibit MRGPRX2-mediated, non-IgE-dependent MCs degranulation as a novel strategy for treating chronic pruritus in AD (156).

EVO756, a selective MRGPRX2 antagonist, potently inhibits SP and compound 48/80-induced histamine release, CD63 upregulation, and cytokine secretion (IL-8, TNF-α) in human LAD2 cells and primary cutaneous MCs, without affecting IgE–FcϵRI-dependent activation (157, 158). It also reprograms MCs transcriptional profiles from an activated to a quiescent state (158) and reduces vascular permeability in humanized mouse models (157), suggesting both anti-inflammatory and barrier-protective effects relevant to AD. EP262, another MRGPRX2 antagonist, has demonstrated both *in vitro* and *in vivo* inhibition of MCs degranulation and vascular permeability by suppressing the release of β-hexosaminidase, tryptase, histamine, IL-6, TNF-α, and other proinflammatory mediators (159, 160). Another lead compound, GE1111, demonstrated efficacy in a DNFB-induced mouse model of AD, ameliorating erythema, scaling, and skin thickening confirmed by histological analysis.

This antagonist also downregulated inflammatory mediators such as Il-1β, Il-13, and TSLP, while upregulating barrier proteins including claudin-1. In a human three-cell co-culture system consisting of MCs, keratinocytes, and macrophages, GE1111 effectively inhibited mast cell–mediated cytokine release, underscoring its dual anti-inflammatory and barrier-restorative potential as an MRGPRX2-targeted therapy (161).

By contrast, C9 represents a novel inverse agonist that stabilizes the inactive conformation of MRGPRX2, thereby reducing its constitutive activity. C9 potently inhibits SP- and compound 48/80-induced degranulation without affecting IgE–FcϵRI-mediated responses and having no effect on murine MrgprB2-expressing MCs, indicating high pathway specificity and cross-species selectivity (162). Although *in vivo* validation and clinical translation of C9 remain pending, its mechanism offers a promising approach to selectively target non-histaminergic itch pathways.

### 5.3 TRPV1 and TRPA1: mechanistically validated targets

TRPV1 and TRPA1 channels are pivotal mediators in itch signal transduction within the mast cell–neuron axis (124). A growing body of experimental and clinical evidence has demonstrated the therapeutic potential of TRP channel antagonists in modulating both pruritus and inflammation in AD. These agents represent a promising class of precision therapeutics that directly target neuroimmune pathways and may offer a breakthrough in AD management.

The TRPA1 antagonist HC-030031 has been shown to significantly reduce spontaneous scratching behavior, epidermal hyperplasia, and dermal thickening in a DNCB-induced mouse model of AD. Treatment also led to decreased MCs infiltration and downregulation of inflammatory cytokines such as IL-4 and IL-13, indicating its potential to alleviate chronic skin inflammation and itch, possibly via non-histaminergic pathways (105). Carboxamide-based TRPA1 antagonists represent a novel class of topical agents with favorable skin permeability and pharmacokinetic properties. In guinea pig models, these compounds significantly reduced cinnamaldehyde-induced neurogenic skin flare, supporting their potential for further development as TRPA1-targeted therapies (163). While TRPA1 antagonists have shown consistent anti-inflammatory and antipruritic effects in preclinical studies, the current pipeline lacks candidates with both high selectivity and optimal pharmacokinetics, and clinical development remains in its early stages (164).

For TRPV1, Asivatrep (PAC-14028) is currently the most clinically advanced small-molecule antagonist. It is the first topical TRPV1-targeting compound of its class to reach phase III trials. In a phase IIb study, Asivatrep significantly reduced pruritus with a 2.3-point decrease in visual analog scale (VAS) scores (165). In the subsequent phase III trial, it achieved a 44.3% mean improvement in EASI and a 36.0% IGA 0/1 response rate, indicating both antipruritic and anti-inflammatory efficacy compared to placebo (166).In parallel, traditional herbal formulations such as Zhenxin Anshen formula have been shown in both AD mouse models and *in vitro* systems to suppress TRPV1 and TRPA1 expression, reduce scratching behavior, and downregulate inflammatory mediators including IgE, IL-4, IL-5, IL-13, and TSLP. These findings highlight its potential as a multi-targeted therapeutic strategy through integrated modulation of neural and immune pathways (167). Although TRPV1 antagonists show promising antipruritic efficacy, their further development has been limited by safety concerns. As TRPV1 is also involved in thermoregulation, early-phase clinical trials have reported adverse events including hyperthermia and hypothermia, raising challenges for the long-term use of this target (168).

### 5.4 Other targets

Beyond direct antagonism of MRGPRX2, several studies have explored indirect strategies to suppress its activity through upstream or parallel regulatory mechanisms.

Siglec-8 is an inhibitory receptor selectively expressed on human MCs and eosinophils. It exerts broad immunomodulatory effects by interfering with downstream signaling pathways of FcϵRI, including Syk, ERK, and Akt (169). In transgenic mice expressing human Siglec-8, treatment with an anti-Siglec-8 monoclonal antibody significantly suppressed IgE-mediated MCs degranulation and effectively blocked MRGPRX2-mediated, non-IgE-dependent MCs activation and pruritus (95). These findings suggest that Siglec-8 may serve as a dual-pathway immunoregulatory target with broad therapeutic potential in neuroimmune-mediated disorders. GluK2, a kainate-type glutamate receptor subunit predominantly expressed in MCs, forms functional complexes with kainate receptor subunit 2 (KA2) subunits. This receptor senses glutamate released from peripheral neurons and negatively regulates MrgprB2-mediated MCs activation, thereby serving as a key suppressor of neuroimmune hyperactivation and maintaining local immune homeostasis (115). *In vitro* mouse MCs, *ex vivo* human skin explants, and *in vivo* murine models have demonstrated that pharmacological activation of GluK2 significantly inhibits MCs degranulation as well as cutaneous inflammatory and pruritic responses, supporting its potential as a negative regulatory therapeutic target (138).

The calcium release–activated calcium (CRAC) channels formed by calcium release-activated calcium channel protein (ORAI) 1 and ORAI2 also play essential roles in the non-IgE-dependent activation of MCs within the neuroimmune axis, and they are increasingly being investigated as novel pharmacological targets (170, 171). *In vitro* studies have shown that activation of MRGPRX2 or its murine ortholog MrgprB2 induces MCs degranulation via Orai channels, with Orai1 and Orai2 being the predominant contributors. Importantly, Orai2 also acts as a negative regulator, as its genetic deletion augments CRAC currents, enhances MCs degranulation, and exacerbates anaphylaxis severity (172). In contrast, silencing ORAI1/2 with shRNA or pharmacological inhibition using the CRAC channel blocker Synta66 markedly suppressed SP-induced Ca²^+^ influx, CD63 expression, β-hex release, and production of TNF-α, IL-8, and CCL3, while reducing ERK1/2 and Akt phosphorylation. *In vivo*, Synta66 attenuated SP-induced vascular permeability and neutrophil recruitment, highlighting the central role of ORAI channels in MRGPRX2/MrgprB2-driven neuroimmune amplification (173). Further development led to the generation of DS-2741a, a monoclonal antibody that selectively targets ORAI1. In human ORAI1 knock-in mice, DS-2741a inhibited T cell activation and MCs degranulation, and alleviated house dust mite–induced dermatitis, supporting the therapeutic potential of ORAI1 blockade in allergic diseases (174).

In addition to synthetic compounds, several natural products have gained attention for their ability to regulate MCs function. Flavonoids such as myricetin and the diterpenoid dehydroandrographolide (DA), both derived from medicinal plants, have been shown to activate the inhibitory receptor CD300f and its downstream Src homology region 2 domain-containing phosphatase (SHP)1/2 signaling cascade. These pathways negatively regulate MRGPRX2-mediated MCs degranulation, resulting in reduced histamine release, suppression of proinflammatory cytokines, and attenuation of vascular permeability and pseudo-allergic skin reactions in LAD2 human MCs and murine models (175, 176). These findings underscore their potential as non-IgE-targeting anti-inflammatory candidates. Celastrol has also been shown to suppress the expression of MRGPRX2, ORAI1, and ORAI2. In DNFB-induced mouse models of AD, Celastrol reduced MCs activation, skin inflammation, and histamine release, while in compound 48/80-induced models it alleviated pruritic behavior. Its anti-inflammatory and antipruritic effects were significantly diminished in MRGPRX2-overexpressing mice, indicating that its primary mechanism involves modulation of the MRGPRX2/ORAI1/ORAI2 axis (177).

## 6 Therapeutic challenges and perspectives

Although the mast cell–neuron axis has been increasingly recognized as a central integrative hub that orchestrates neuronal excitation, immune amplification, and sustained inflammation. However, therapeutic strategies targeting this axis continue to face multiple challenges, including limited availability of validated targets, substantial disease heterogeneity, fragmentation of current intervention approaches, and the pressing need to shift from localized symptom control toward comprehensive restoration of systemic immune and neurophysiological homeostasis.

### 6.1 Limitations of current targeted strategies

The IL-31/IL-31RA axis represents one of the most clinically advanced antipruritic targets. Agents such as nemolizumab have demonstrated significant efficacy in alleviating chronic itch in patients with AD (178, 179). However, their primary mechanism of action involves inhibition of neuronal signal transmission at the peripheral nerve terminals, with limited direct effects on skin barrier restoration and local immune modulation. Consequently, these therapies may fall short in addressing the multifaceted pathogenesis of AD, including epidermal repair, microbiome rebalancing, and immune reprogramming, thereby limiting their capacity to achieve sustained disease control throughout the entire disease course (25, 180). In addition, the IL-31/IL-31RA axis contributes not only to pruritus signaling but also to the immunoregulatory balance by inducing the neurogenic release of CGRP, which negatively regulates Th2 responses. Complete blockade of this pathway may provide effective relief from itch but could potentially disrupt immune homeostasis, raising concerns about immune rebound and proinflammatory flare-ups (24).

MRGPRX2, a receptor selectively expressed in primates, presents further challenges due to the absence of a natural ortholog in rodents, which complicates the development of translational animal models. Although MrgprB2 is widely used as the murine homolog, it differs from MRGPRX2 in ligand specificity, signaling potency, and regulatory control (88, 181). As a result, most current evidence is derived from *in vitro* or rodent studies, and preclinical data remain limited. Pharmacological agents targeting MRGPRX2 are still in early stages of development and lack robust translational validation (33).

TRP channels such as TRPV1 and TRPA1 have shown promising antipruritic effects in preclinical models and are considered key targets for neurogenic itch. However, their essential physiological roles in thermoregulation and nociception pose significant safety concerns. Systemic modulation of TRP channels is often associated with adverse effects such as thermal dysregulation and burning sensations, which substantially limit their clinical utility and broader therapeutic application (182, 183).

In addition, the P2X3 receptor, an ATP-gated ion channel expressed on sensory neurons, has attracted growing attention in the context of neurogenic itch. Several novel antagonists have demonstrated potent inhibitory activity in sensory neurons (184). Among them, the representative agent gefapixant has undergone extensive clinical evaluation in chronic cough: a phase IIb trial demonstrated that oral administration of 50 mg twice daily significantly reduced cough frequency (185), and subsequent global phase III studies further confirmed the efficacy and safety of the 45 mg twice-daily regimen (186). Although P2X3 antagonists have shown success in other neurogenic disorders, their investigation in AD remains at an early exploratory stage, and further studies are required to validate their therapeutic potential.

### 6.2 Molecular heterogeneity–driven precision stratification

Although treatment of moderate-to-severe AD has entered a new era centered on biologic agents, interindividual differences in the expression of key neuroimmune genes such as IL-31, MRGPRX2, TRPA1, NGF, and CGRP contribute to molecular heterogeneity that is closely associated with the intensity of pruritus and disease chronicity, and these differences significantly affect patients’ responses to therapy (187). In current clinical practice, the use of biologic agents largely relies on empirical judgment rather than stratification based on immunological characteristics, resulting in a trial-and-error approach to treatment that often delays optimal intervention (188). This, to some extent, accounts for the substantial variability in patient responses to different targeted therapeutic strategies (189, 190). Therefore, there is an urgent need to establish a more refined and mechanism-driven classification system to guide individualized treatment and inform drug development strategies, ultimately improving therapeutic efficacy.

Although several studies have attempted to classify patients with AD based on clinical features and immunological endotypes, these classification frameworks have not yet evolved into standardized tools widely applicable in clinical practice (191).

To improve stratification, a two-dimensional model incorporating pruritus intensity and lesional severity has been proposed by some researchers, with the subtype characterized by severe itch and mild-to-moderate lesions (SI-ML) capturing the discordance between subjective symptoms and objective signs and supporting more targeted and individualized treatment approaches (192). To further address the challenges of disease stratification and mechanistic heterogeneity, cutting-edge technologies such as single-cell RNA sequencing (scRNA-seq), spatial transcriptomics, artificial intelligence algorithms, and integrative multi-omics analysis have enabled the high-resolution identification of key cellular populations and molecular markers that are closely associated with disease severity and therapeutic response (189, 193–195). These approaches facilitate the prediction of individual responsiveness to specific targeted therapies, including IL-4Ra, IL-13, and IL-31 inhibitors. In clinical practice for psoriasis, a study established an *in vitro* model that combines peripheral blood mononuclear cells (PBMCs) from patients with streptococcal stimulation to mimic immune activation. The results demonstrated that levels of IFN-γ, IL-17A, and their ratios to IL-4/IL-13 could effectively predict the therapeutic efficacy of multiple biologic agents (196). These findings offer valuable insights that may inform immunological stratification and targeted treatment strategies in AD.

In summary, the application of high-resolution technologies to systematically delineate immunophenotypic differences among patients and to develop classification models that are closely associated with therapeutic outcomes, particularly those focusing on the functional activity of critical pathways such as the mast cell–neuron axis, will facilitate the implementation of more efficient, individualized, and low-toxicity targeted therapies, while also advancing drug development and clinical translation.

### 6.3 Theoretical and practical insights into multi-target synergistic interventions

Most current therapeutic strategies targeting the mast cell–neuron axis remain focused on single-pathway interventions, primarily involving blockade of specific ligand–receptor interactions. However, the persistence of chronic pruritus in AD is not driven by isolated signaling events but rather results from a complex interplay of neuronal sensitization, structural coupling, and immune dysregulation. Both clinical and experimental studies indicate that although single-target approaches may offer short-term symptomatic relief, they often fail to interrupt the dynamic feedback loop of degranulation, neuronal excitation, and inflammatory amplification. These findings underscore the necessity of coordinated, multi-target intervention strategies to achieve sustained disease control (197).

Therefore, the concept of synergistic blockade across multiple pathways is gradually replacing the traditional single-target paradigm. Simultaneous targeting of inflammatory mediators and neuronal signaling components enables cascade-level inhibition across different layers of the neuroimmune axis, including receptor-level, ion channel-level, and structural domains. This multi-tiered approach facilitates integrated modulation from local barrier function to systemic immune response and represents a conceptual shift from discrete ‘point inhibition’ to coordinated ‘axis regulation’ in the treatment of chronic pruritus (197, 198).

Within this context, traditional Chinese medicine (TCM) offers unique advantages due to its intrinsic multi-target and low-toxicity properties. Herbal formulas and their active constituents frequently modulate multiple molecular targets and signaling cascades associated with AD pathogenesis, thereby embodying a systems-level therapeutic approach (199, 200). For instance, natural compounds such as myricetin and dehydroandrographolide have been shown to negatively regulate MRGPRX2-mediated MCs degranulation, and may hold promise for further exploration into additional neuroimmune regulatory mechanisms (175, 176). Moreover, myricetin can also effectively alleviates erythema, edema, pruritus, and epidermal thickening by suppressing proinflammatory cytokines, modulating Th1/Th2/Th17 immune balance, inhibiting MCs infiltration and the release of histamine and IgE, and restoring skin barrier function through the inhibition of NF-κB and STAT1 signaling pathways *in vivo*/*in vitro* (201, 202). In addition, the TCM Jiu-Wei-Yong-An Formula has been shown to reduce IL-31 expression in the skin of AD mice, while also alleviating cutaneous inflammation, pruritic behavior, epidermal hyperplasia, MCs infiltration, and dysregulated Th1/Th2 cytokine expression, exerting synergistic anti-inflammatory and immunomodulatory effects primarily through inhibition of the JAK1/STAT3 and MAPK signaling pathways (203).

Taken together, these insights underscore the necessity and feasibility of adopting multi-target, systems-oriented strategies for the treatment of chronic pruritus in AD. Such approaches offer clear advantages in disrupting complex neuroimmune networks, and align closely with the intrinsic therapeutic potential of traditional Chinese medicine, which operates through coordinated, network-based mechanisms.

## 7 Conclusion

The mast cell–neuron axis functions as a key neuroimmune interface that contributes to the initiation, progression, and persistence of chronic pruritus in AD. It mediates both sensory neuronal activation and immune amplification and establishes a self-sustaining itch–inflammation loop through complex signaling pathways and intercellular interactions. This dynamic network makes chronic itch in AD difficult to effectively control.

Current therapeutic approaches targeting this axis, particularly IL-31RA inhibitors, have demonstrated partial success. These treatments modulate specific signaling pathways, leading to relief of itch symptoms and improved quality of life. However, the development of drugs targeting other key components, including MRGPRX2, TRPV1/TRPA1, ORAI1/2/3 and P2X3 receptors, is still constrained by species specificity, safety concerns, and the lack of validated translational models. As a result, their clinical application remains limited, and no breakthrough has yet been achieved.

The complexity of AD, including its immunological heterogeneity, multilayered mast cell–neuron interactions, and the finely regulated nature of the neuroimmune axis, presents further challenges for therapeutic translation. Clinical limitations such as suboptimal efficacy, variability in patient response, risk of immune imbalance, and concerns regarding long-term safety continue to hinder progress. Future research should focus on elucidating the precise mechanisms through which the mast cell–neuron axis contributes to chronic pruritus and identifying effective multi-target strategies that can comprehensively regulate this axis. Advances in technologies such as single-cell sequencing, spatial transcriptomics, and integrative multi-omics are expected to facilitate the identification of pathogenic pathways and functional patient subtypes, supporting the development of personalized therapeutic strategies. In parallel, traditional medicine, particularly Chinese herbal medicine, may offer additional therapeutic value due to its multi-target and systemic regulatory properties. Investigating its modulatory effects on the mast cell–neuron axis may help establish safe and effective treatment options tailored to individual patient profiles.

In conclusion, continued research into the mast cell–neuron axis will provide a strong foundation for the precision management of chronic pruritus in AD and may contribute to long-term symptom control and improved patient outcomes.
